# Correlation of clinical pigmentary characteristics with histopathologically-confirmed dysplastic nevi in nonfamilial melanoma patients. Studies of melanocytic nevi IX.

**DOI:** 10.1038/bjc.1991.431

**Published:** 1991-11

**Authors:** G. C. Roush, R. L. Barnhill

**Affiliations:** Cancer Prevention Research Institute, New York, NY.

## Abstract

The dysplastic melanocytic nevus (DMN) is the key clinical marker for the familial dysplastic nevus syndrome and has also been associated with high risk for non-familial melanoma. Characterisations of DMN itself have been qualitative and on a case-by-case basis. In this study, we provided clinical and histological characterisations for each of 150 pigmented lesions from 150 patients with prior malignant melanoma. The steps involved in the study were as follows: (1) The two to four clinicians characterised pigmented lesions on each of 150 patients, and the lesion closest in characteristics to an atypical nevus was quantitatively described based on size, border characteristics and colour characteristics; (2) The lesion was then removed and independently quantified by a single dermatopathologist without knowledge of the clinical features; (3) We computed the correlation between each of the clinical variables and each of the histologic features for each of the 150 patients. Histologic diagnosis of dysplastic nevus was strongly associated with total number of palpable arm nevi, total number of any arm nevi, total number of nevi on the body of any type, and total number of clinically atypical nevi on the body (correlation coefficients 23.2% to 30.4% with P less than 0.01 in each instance). There were also strong correlations between the counts of numbers of nevi and certain types of architectural histologic features, including fusion (bridging of junctional nests), lymphocyte response and fibroplasia of the papillary dermis. Histologic evaluation of solar elastosis was negatively correlated with total numbers of nevi and total number of clinically atypical nevi (P less than 0.01). Freckling on forearm and on shoulders showed no significant positive or negative correlations with any of the histologic features nor with overall diagnosis of dysplastic nevus. We conclude that observations regarding total numbers of nevi (either normal or clinically atypical nevi) are correlated with nuclear and architectural histologic dysplasia on biopsy of the most atypical pigmented lesions.


Br. J. Cancer (1991), 64, 943-947 © Macmillan Press Ltd., 1991

### Correlation of clinical pigmentary characteristics with histopathologically-confirmed dysplastic nevi in nonfamilial melanoma patients. Studies of melanocytic nevi IX

G.C. Roush[1] & R.L. Barnhill[2]

[1]*Cancer Prevention Research Institute, 36 East 22nd Street, New York, NY;* [2]*Dermatopathology Division and Dermatology Service, Massachusetts General Hospital and Harvard Medicine School, Boston, Massachusetts, USA.*

**Summary** The dysplastic melanocytic nevus (DMN) is the key clinical marker for the familial dysplastic nevus syndrome and has also been associated with high risk for non-familial melanoma. Characterisations of DMN itself have been qualitative and on a case-by-case basis. In this study, we provided clinical and histological characterisations for each of 150 pigmented lesions from 150 patients with prior malignant melanoma. The steps involved in the study were as follows: (1) The two to four clinicians characterised pigmented lesions on each of 150 patients, and the lesion closest in characteristics to an atypical nevus was quantitatively described based on size, border characteristics and colour characteristics; (2) The lesion was then removed and independently quantified by a single dermatopathologist without knowledge of the clinical features; (3) We computed the correlation between each of the clinical variables and each of the histologic features for each of the 150 patients.

Histologic diagnosis of dysplastic nevus was strongly associated with total number of palpable arm nevi, total number of any arm nevi, total number of nevi on the body of any type, and total number of clinically atypical nevi on the body (correlation coefficients 23.2% to 30.4% with $P<0.01$ in each instance). There were also strong correlations between the counts of numbers of nevi and certain types of architectural histologic features, including fusion (bridging of junctional nests), lymphocyte response and fibroplasia of the papillary dermis. Histologic evaluation of solar elastosis was *negatively* correlated with total numbers of nevi and total number of clinically atypical nevi ($P<0.01$). Freckling on forearm and on shoulders showed no significant positive or negative correlations with any of the histologic features nor with overall diagnosis of dysplastic nevus.

We conclude that observations regarding total numbers of nevi (either normal or clinically atypical nevi) are correlated with nuclear and architectural histologic dysplasia on biopsy of the most atypical pigmented lesions.

Pigmentary characteristics, such as freckling (Dubin *et al.*, 1986; Elwood *et al.*, 1986; Green *et al.*, 1985*a*; Klepp & Magnus, 1979), numbers of nevi (Dubin *et al.*, 1986; Elwood *et al.*, 1986; Green *et al.*, 1985*a*; Holman & Armstrong, 1984*b*; Beral *et al.*, 1983; Hicks *et al.*, 1985; Swerdlow *et al.*, 1986), and gross morphologic abnormalities of nevi (Swerdlow *et al.*, 1986; MacKie *et al.*, 1989) have been associated with increased risk for the development of primary cutaneous malignant melanoma. The dysplastic melanocytic nevus (DMN) is thought to be a direct precursor of familial melanoma (Reimer *et al.*, 1978; Clark *et al.*, 1978; Greene *et al.*, 1985) and, for more common forms of melanoma, DMN may be a direct precursor or perhaps a marker of increased risk for melanoma (Swerdlow *et al.*, 1986; MacKie *et al.*, 1989; Rhodes *et al.*, 1980; Nordlund *et al.*, 1985; Holly *et al.*, 1987; Roush, 1988). Therefore, we hypothesised that other pigmentary features associated with risk for melanoma, i.e. freckling, numbers of nevi, might correlate with histologic dysplasia in clinically atypical nevi from melanoma patients. Quantification of the strength of this putative association might indicate which pigmentary features could potentially augment or reduce suspicion of a DMN on clinical assessment of the skin. Such results could have etiologic implications. Here, we report the extent of correlation of several pigmentary features with histologic melanocytic dysplasia and with other histologic features of the most atypical lesion removed from each of 150 patients with non-familial melanoma.

### Material and methods

One hundred and fifty-three newly-diagnosed melanoma patients participated in the study and were examined in the Yale Melanoma Unit from 1 January 1983 to 1 July 1987. Participants were referred to the Yale Melanoma Clinic for evaluation and treatment primarily from the southern Connecticut region. Criteria for enrollment in the study included newly-diagnosed malignant melanoma, non-Hispanic caucasian subjects, age limitation: above 20 and under 70 years of age, and absence of family history of melanoma in two or more first degree relatives. Patients with these criteria were invited to enrol in the study and informed consent was obtained. The proportion participating exceeded two-thirds for all eligible patients evaluated during this period.

As described previously, all study subjects underwent an epidemiologic interview concerning occupation and sun exposure variables, exam of the entire skin excluding the genitalia, and removal of at least one (and usually two) pigmented lesions for histopathologic examination. The comprehensive skin examination included an assessment of freckling characteristics; a count of palpable arm nevi below the level of the axillae (nevi could be of any size); a count of any nevi on the arms, palpable or not; a count of total body nevi greater than 3 mm in greatest diameter; a count of the total number of atypical nevi; and a quantification of the morphology and colour of the most atypical nevi. Freckles were defined as light-tan to brown, completely macular lesions without any surface change or distortion of skin cleavage lines and generally measuring from 2–3 mm to 10 mm in diameter. Melanocytic nevi were defined as relatively flat (but associated with accentuation of skin cleavage lines) or raised lesions, generaly measuring greater than 3 to 5 mm in diameter, and pink, flesh-coloured, or pigmented. Distinction of a junctional nevus from a simple lentigo is difficult. In our experience most lentigines are macular, do not exhibit distortion of skin cleavage lines, and measure 2–3 mm in diameter (but may be larger). Junctional nevi, on the other hand, are frequently slightly palpable, exhibit distortion of skin cleavage lines, and usually measure greater then 3 mm in size. Definitive classification is by histological examination. Nevi were further defined as not being obvious seborrheic keratoses, solar lentigines, warts, or dermatofibromas. The designation of a nevus as atypical was based on the subjective

Correspondence: R.L. Barnhill, Warren 827, Dermatopathology Division, Massachusetts General Hospital, Boston, MA 02114, USA.
Received 29 November 1990; and in revised form 11 July 1991.

assessment of each individual examiner but generally was directly related to the number (usually at least three or more) of gross morphological features outlined below, e.g. size greater than 5 mm, irregular border, haphazard colour. If any nevi were present, the clinical characteristics of up to eight of the most atypical nevi were recorded.

*Clinical features evaluated*

The following clinical characteristics were correlated with histomorphological features:

(1) Estimation of freckling on shoulders: scored as less than 20 freckles, 20 to 50 freckles, and greater than 50 freckles;
(2) Estimation of freckling on right forearm as categorised above;
(3) Number of palpable arm nevi below level of axillae of any size;
(4) Number of nevi on arms below level of axillae, palpable or not, of any size;
(5) Total number of nevi on body greater than 3 mm in greatest diameter;
(6) Total number of atypical nevi on body.

Each patient was independently examined by two to four physicians. The number of patients assessed by each examiner varied from 76 to 148, with nearly half the patients evaluated by three physicians. Variation in the number of nevi analysed in the study was also dependent on the presence of a complete set of observations for each lesion. Some of the clinical and histologic categories were not incorporated until after the study had been begun (e.g. freckling, total number of any arm nevi, and prominent vascularity).

In addition, each examiner recorded for quantification on examination sheets, the individual characteristics of up to eight of the most atypical lesions. These characteristics included longest diameter; whether the lesion was macular, papular or both; clinical appearance of asymmetry; irregular border; predominant colour; and haphazard colouration. Based on this independently derived assessment, a consensus was reached among examiners as to the clinically most atypical lesion, which was then designated, photographed, and removed for histopathologic exam by simple excision or deep saucerisation technique. The designation of 'the most atypical lesion' was related to the greatest number of abnormal lesion characteristics. No particular parameter was more heavily weighted relative to the other clinical characteristics, including size. It should be emphasised that the most atypical lesion was not necessarily clinically atypical, an atypical nevus or a nevus at all because some patients had only typical-appearing nevi or no nevi on their cutaneous surface (i.e., the most atypical lesion thus could have been a dermal nevus, lentigo, or dermatofibroma).

The results of the histologic evaluation of the lesions removed have been previously reported in detail and are not the subject of this communication (Barnhill *et al.*, 1990). In brief, 17% of the lesions removed were diagnosed as dysplastic nevi histologically based on the presence of discontinuous nuclear atypia of intraepidermal nevo-melanocytes and well-recognised architectural features. Nuclear atypia was defined as at least 10–50% of intraepidermal nevomelanocytes exhibiting at least three of the four following characteristics: (1) nuclear enlargement; (2) nuclear pleomorphism; (3) nuclear hyperchromatism, and (4) prominent nucleoli.

The histologic features assessed in study have been previously defined (Barnhill *et al.*, 1990) and included architectural and cytologic (nuclear and cytoplasmic) characteristics, as listed in Table I.

*Statistical analysis*

Spearman's rank correlation coefficient (Zar, 1984) was used to measure the association between each of the individual clinical features and each of the histomorphological variables. This was preferred over least squares because it is a nonparametric statistic that may be applied when data are not normally distributed.

### Results

Table I provides summary statistics for each of the clinical and histologic features. The magnitude of the standard deviation may be compared to the mean to obtain an idea of the dispersion for each variable. The variable for freckling may have somewhat less heterogeneity and therefore may have less potential for discrimination.

*Relationship between freckling and histology of clinically most atypical nevus*

As shown in Table II, degree of freckling was not associated with a histologic diagnosis of DMN or any of the histologic features. Similarly, there was no relationship between freckling when assessed in three categories *vs* the histologic diagnosis of the most atypical nevus (Table IIIa).

*Relationship between numbers of nevi and histology of clinically most atypical nevus*

DMN was correlated strongly with all counts of nevi, including palpable arm nevi (23.2%, $P<0.01$), total arm nevi (30.4%, $P<0.01$), total body nevi greater than 3 mm (29.4%, $P<0.001$), and total atypical nevi (28.9%, $P<0.001$) (Table II).

With regard to the individual histologic features, the total number of atypical nevi showed a significant correlation with 16 of 18 histologic features including all nuclear abnormalities. Total body nevi correlated significantly with 12 histological features, including the abnormal nuclear features. Total arm nevi correlated with 13 histologic features and palpable arm nevi correlated with 11 histological features, including most of the nuclear parameters.

Solar elastosis was *negatively* correlated with each of the six counts of pigmentary characteristics. The associations were −30.1% and −21.7% ($P<0.01$ in each instance) for total numbers of nevi and for total number of clinically atypical nevi, respectively.

Cross classification of numbers of nevi as categorical variables *vs* the histology of the most atypical nevus revealed statistically significant associations for all four counts of nevi (i.e. palpable arm nevi, any arm nevi, total body nevi, total atypical nevi) (Table IIIb).

### Discussion

Our results have indicated that numbers of melanocytic nevi – whether total body, on the arms, or having clinical atypicality – are strongly correlated with the finding of histologic melanocytic dysplasia and with many of the histologic features of dysplastic nevus. On the other hand, there was no relationship between the tendency to freckle and dysplastic nevus confirmed on histologic examination.

At least two previous studies have documented an association between numbers of nevi and *clinically* atypical nevi (Holly *et al.*, 1987; Roush, 1988). To our knowledge, the present study is the first to quantify an association between numbers of nevi and confirmation of histologically dysplastic nevus. Further, these data indicate that different types of nevus counts are similarly correlated with confirmation of a dysplastic nevus. Numbers of arm nevi have been associated with increased risk for melanoma, and therefore the associations between arm nevi and histologic dysplasia in this study lend further credibility to these studies. Finally, the present study was unique in its correlations of nevus counts with individual histologic features. Of particular interest was the correlation of numbers of nevi with nuclear atypicality and host response, especially lymphocytic infiltrates and fibroplasia.

Also of interest was the negative correlation of solar elastosis with total numbers of nevi, both typical and atypical. At the same time, there was no correlation between solar elastosis and the presence of nevi on the arms. Solar elastosis

**Table I** Descriptive statistics for clinical and histologic features

| *Clinical variables* | | | *Category* | | | |
|---|---|---|---|---|---|---|
| Freckling on forearms | n | <20 | 20–50 | >50 | Total | |
| | 104 | 47.1% | 17.3 | 35.6 | 100% | |
| Freckling on forearms | n | <20 | 20–50 | >50 | Total | |
| | 104 | 19.2% | 23.1 | 57.7 | 100% | |
| | *n* | *Median* | *Mean* | *Std Dev* | *Min* | *Max* |
| Palpable arm nevi | 152 | 2.0 | 4.0 | 5.1 | 0 | 37 |
| Any arm nevi | 84 | 3.0 | 5.6 | 7.4 | 0 | 38 |
| Total nevi, 3.0 mm+ in diameter | 150 | 13.0 | 20.3 | 23.6 | 1 | 170 |
| Total atypical nevi | 150 | 1.0 | 2.0 | 3.2 | 0 | 22 |

| *Histopathology of the most atypical nevus* | | | *Category* | | |
|---|---|---|---|---|---|
| *Architectural features:* | *n* | *0* | *1* | *2* | *3* |
| Asymmetry (0,1)[a] | 128 | 71.1% | 28.9% | N/A | N/A |
| Lateral extension or poor circumscription (0,1) | 140 | 26.4% | 73.6% | N/A | N/A |
| Lentiginous hyperplasia of epidermis (0,1) | 148 | 9.5% | 90.5% | N/A | N/A |
| Basal melanocytic hyperplasia (0,1,2) | 148 | 8.8% | 40.5% | 50.7% | N/A |
| Junctional nesting disarray (0,1) | 147 | 68.7% | 31.3% | N/A | N/A |
| Fusion (bridging) (0,1) | 138 | 71.0% | 29.0% | N/A | N/A |
| Suprabasal melanocytes (0,1) | 146 | 87.0% | 13.0% | N/A | N/A |
| Lymphocystic response (0,1,2) | 149 | 32.2% | 59.7% | 8.1% | N/A |
| Melanophages (0,1) | 145 | 34.5% | 65.5% | N/A | N/A |
| Fibroplasia of papillary dermis (0,1,2) | 146 | 45.2% | 48.6% | 6.2% | N/A |
| Prominent vascularity (0,1) | 112 | 58.0% | 42.0% | N/A | N/A |
| Solar elastosis (0,1) | 146 | 82.9% | 15.8% | 1.4% | N/A |
| *Nuclear features:* | *n* | *0* | *1* | *2* | *3* |
| Nuclear enlargement (0,1,2,3) | 157 | 75.8% | 5.1% | 16.6% | 2.5% |
| Nuclear pleomorphism (0,1,2,3) | 157 | 63.1% | 3.2% | 31.2% | 2.5% |
| Nuclear hyperchromatism (0,1,2,3) | 157 | 78.3% | 3.8% | 15.3% | 2.5% |
| Prominent nucleoli (0,1,2,3) | 155 | 90.3% | 3.9% | 4.5% | 1.3% |
| *Cytoplasmic features:* | *n* | *0* | *1* | *2* | *3* |
| Abundant pale or esoinophilic cytoplasm (0,1,2,3) | 150 | 86.7% | 2.0% | 11.3% | 0% |
| Dusty cytoplasm (0,1,2,3) | 150 | 84.0% | 4.7% | 10.7% | 0.7% |
| Melanosomes (Large melanin granules) (0,1,2,3) | 150 | 62.0% | 4.0% | 34.0% | 0% |

[a]Numbers in parentheses indicate categories for each variable (see Materials and methods) or the absolute number of nevi for each variable. Histologic variables were generally scored as absent = 0 or present = 1. For additional explanation of histologic variables, refer to Barnhill, Roush & Duray, 1990.

**Table II** Correlation of individual clinical features with histological diagnosis of dysplastic nevus and individual histologic features expressed as Spearman correlation coefficient (per cent)

| | *Clinical features* | | | | | |
|---|---|---|---|---|---|---|
| *Histologic features* | *Freckling on shoulders* | *Freckling on right arm* | *Total palpable arm nevi* | *Total arm nevi* | *Total nevi greater than 3 mm* | *Total atypical nevi* |
| Histologic diagnosis dysplastic nevus[a] | 3.8 (104)[e] | 0.0 (104) | 23.2 (152)[c] | 30.4 (84)[c] | 29.4 (150)[d] | 28.9 (150)[d] |
| Asymmetry | 19.6 (83) | −1.8 (83) | 23.1 (124)[c] | 24.1 (68)[b] | 1.5 (123) | 18.6 (122)[b] |
| Lateral extension | 10.6 (91) | 1.8 (91) | 20.9 (135)[b] | 23.4 (71) | 18.5 (133)[b] | 31.2 (133)[d] |
| Lentiginous hyperplasia of epidermis | −1.4 (95) | −7.5 (95) | 34.1 (143)[d] | 40.5 (75)[c] | 32.4 (141)[d] | 34.5 (141)[d] |
| Basal melanocytic hyperplasia | −2.0 (96) | −16.4 (96) | 19.4 (143)[b] | 33.5 (76)[c] | 22.1 (141)[c] | 34.0 (142)[d] |
| Junctional nesting disarray | 1.7 (94) | −6.3 (94) | 15.1 (142) | 27.9 (75)[b] | 9.4 (140) | 19.3 (140)[b] |
| Fusion | 0.6 (95) | −3.5 (95) | 12.1 (134) | 29.3 (75)[b] | 21.5 (132)[b] | 31.5 (132)[d] |
| Suprabasal melanocytes | 9.6 (93) | 7.8 (93) | 14.3 (141) | 23.3 (73)[b] | 5.0 (139) | 14.2 (139) |
| Lymphocytic response | 2.2 (96) | 1.5 (96) | 23.3 (144)[c] | 38.6 (76)[d] | 23.9 (142)[c] | 28.4 (142)[d] |
| Melanophages | 16.2 (92) | −15.1 (92) | 14.0 (140) | 14.4 (72) | 9.6 (138) | 12.7 (138) |
| Prominent vascularity | 9.7 (79) | 0.7 (79) | 13.5 (109) | 11.8 (70) | 19.5 (108)[b] | 24.4 (107)[b] |
| Fibroplasia of papillary dermis | 11.2 (94) | −5.8 (94) | 23.8 (141)[c] | 34.6 (74)[c] | 25.3 (139)[c] | 29.8 (139)[d] |
| Solar elastosis | −6.7 (94) | −4.2 (94) | −11.5 (141) | −14.3 (74) | −30.1 (139)[d] | −21.7 (149)[c] |
| Nuclear enlargement | −2.2 (103) | 5.5 (103) | 16.6 (151)[b] | 26.3 (83)[b] | 21.7 (149)[c] | 22.2 (149)[c] |
| Nuclear pleomorphism | 5.5 (103) | 3.6 (103) | 27.6 (151)[d] | 39.9 (83)[d] | 34.4 (149)[d] | 27.7 (149)[d] |
| Hyperchromatism | 6.8 (103) | 6.1 (103) | 21.0 (151)[c] | 28.1 (83)[b] | 25.0 (149)[c] | 27.7 (149)[d] |
| Prominent nucleoli | 9.3 (101) | 8.1 (101) | 10.8 (149) | 18.4 (81) | 18.2 (147)[b] | 22.6 (147)[c] |
| Abundant pale cytoplasm | −10.9 (97) | 1.9 (97) | 7.1 (145) | 10.5 (77) | 18.3 (143) | 19.1 (143) |
| Dusty cytoplasm | 2.6 (97) | −1.7 (97) | 19.7 (145)[b] | 16.7 (77) | 15.6 (143)[b] | 25.2 (143)[c] |
| Large melanin granules | −1.6 (97) | −12.8 (97) | 0.7 (97) | 11.6 (77) | 11.8 (143) | 13.1 (143) |

[a]Referred to in text as 'DMN', Dysplastic Melanocytic Nevus; [b]$P<0.05$; [c]$P<0.01$; [d]$P<0.001$; [e]Number of patients examined in parentheses.

**Table IIIa** Cross classification of histology of the most atypical nevus with freckling on clinical exam

| *Histology of nevus* | *Freckling on shoulders* <20 | 20–50 | 50+ | *Freckling on forearms* <20 | 20–50 | 50+ |
|---|---|---|---|---|---|---|
| Normal | 20.9[a] | 23.9 | 55.2 (n = 67) | 46.3 | 20.9 | 32.8 (n = 67) |
| Architectural dysplasia only | 5.6 | 22.2 | 72.2 (n = 18) | 38.9 | 11.1 | 50.0 (n = 18) |
| Nuclear and architectural dysplasia[b] | 26.3 | 21.0 | 52.6 (n = 19) | 57.9 | 10.5 | 31.6 (n = 19) |
| Total sample: | 104 | | | 104 | | |
| Chi square, DF: | 3.22, 4 | | | 3.42, 4 | | |
| *P* value | 0.523 | | | 0.490 | | |

[a]These numbers represent percentages in each of the three categories. Parentheses indicate row total. [b]Referred to in text as 'DMN', Dysplastic Melanocytic Nevus.

**Table IIIb** Cross classification of histology of the most atypical nevus with numbers of nevi on clinical exam

| *Histology of nevus* | *Palpable arm nevi* 0,1 | 2–4 | 5–37 | *Any arm nevi* 0,1 | 2–5 | 6–38 | *Total nevi on body* 0–7 | 8–20 | 21–170 | *Total atypical nevi* 0 | 1–2 | 3–22 |
|---|---|---|---|---|---|---|---|---|---|---|---|---|
| Normal | 44.2[a] | 35.6 | 20.2 (n = 104) | 37.5 | 42.9 | 19.6 (n = 56) | 38.8 | 37.9 | 23.3 (n = 103) | 53.9 | 23.5 | 22.6 (n = 102) |
| Architectural dysplasia only | 16.0 | 28.0 | 56.0 (n = 25) | 15.4 | 30.8 | 53.9 (n = 13) | 20.8 | 20.8 | 58.3 (n = 24) | 20.0 | 48.0 | 32.0 (n = 25) |
| Nuclear and architectural dysplasia | 30.4 | 30.4 | 39.1 (n = 23) | 26.7 | 20.0 | 53.3 (n = 15) | 13.0 | 43.5 | 43.5 (n = 23) | 26.1 | 39.1 | 34.8 (n = 23) |
| Total sample: | 152 | | | 84 | | | 150 | | | 150 | | |
| Chi square, DF: | 14.97, 4 | | | 10.55, 4 | | | 15.60, 4 | | | 13.49, 4 | | |
| *P* value: | 0.005 | | | 0.032 | | | 0.004 | | | 0.009 | | |

[a]These numbers represent percentages in each of the three categories. Parentheses indicate row total. [b]Referred to in text as 'DMN', Dysplastic Melanocytic Nevus.

is thought to reflect chronic solar damage of the skin on portions of the skin 'doubly exposed'. The negative relationships observed here could potentially be explained by anatomic site alone, i.e., nevi may occur more commonly on the trunk where coincidentally solar exposure and hence solar elastosis are less prominent, differing patterns of sun exposure to different anatomic sites (continuous *vs* intermittent), or accelerated aging associated with solar exposure. The patterns of sun exposure may bear some relationship with the development of nevi (Kopf *et al.*, 1978; Armstrong *et al.*, 1986; Kopf *et al.*, 1985; Kopf *et al.*, 1986). However, further research is needed to verify the validity of these results, particularly the role of anatomic site.

Freckling tendency has been documented in several studies as a risk factor for the development of melanoma (Dublin *et al.*, 1986; Elwood *et al.*, 1986; Green *et al.*, 1985*a*; Klepp & Magnus, 1979; Roush *et al.*, 1987). However, the relationship between freckling and dysplastic nevi has not been investigated. Our findings indicate that there seems to be no relationship between tendency to freckle and histologically confirmed melanocytic dysplasia. In a case-control study of various risk factors for melanoma, MacKie and colleagues found that melanoma risk from freckling was independent of the risk associated with numbers of nevi (MacKie *et al.*, 1989). If freckling is a risk factor for melanoma, our data would suggest that those persons with elevation in risk from freckling are distinct from those with elevation in risk from clinically dysplastic melanocytic nevi.

These data are consistent with etiologic concepts of melanoma with respect to dysplastic nevi as melanoma precursors and with respect to the relatively minor role of freckling as markers of those at risk for malignant melanoma. Relative risks for melanoma in individuals with freckling have generally been elevated in the range of 2- to 4-fold (Elwood *et al.*, 1986; Klepp & Magnus, 1979; Beral *et al.*, 1983; Roush *et al.*, 1987). However, relative risks for both total numbers of nevi of any type and total numbers of atypical nevi have ranged from 3- to 30-fold, with most results being in the area of 5- to 20-fold (Elwood *et al.*, 1986; Holman & Armstrong, 1984*b*; Swerdlow *et al.*, 1986; Rhodes *et al.*, 1980; Nordlund *et al.*, 1985; Holly *et al.*, 1987; Roush, 1988; Roush *et al.*, 1986; Green *et al.*, 1986). Thus, the epidemiologic studies would suggest that nevi (either total nevi or numbers of atypical nevi) are better predictors of melanoma risk than freckling, and these patterns are entirely consistent with the results in Tables II and III.

Misclassification on clinical exam could have a major impact on these inter-relationships. In other analyses, we are examining the relative ease of classification of numbers of nevi and of freckling, because problems in clinical measurement as well as etiologic relationships could explain these patterns.

In conclusion, the findings from the present study provide for the first time quantitative data about the relationships between clinical features such as freckling and numbers of nevi on the one hand, and histologic melanocytic dysplasia. While there was no correlation whatsoever between freckling and histologic dysplasia, all counts of nevi were strongly associated with histologically-confirmed dysplastic nevi. These results should aid in better defining the clinical phenotype of patients with dysplastic nevi and the prediction of histologic dysplasia from clinical features.

**References**

ARMSTRONG, B.K., DEKLERK, N.H. & HOLMAN, C.D.J. (1986). Etiology of common acquired melanocytic nevi: constitutional variables, sun exposure, and diet. *JNCI*, **77**, 329.

BARNHILL, R.L., ROUSH, G.C. & DURAY, P.H. (1990). Correlation of histologic architectural and cytoplasmic features with nuclear atypia in atypical (dysplastic) nevomelanocytic nevi. *Hum. Pathol.*, **21**, 51.

BERAL, V., EVANS, S., SHAW, H. & MILTON, G. (1983). Cutaneous factors related to the risk of malignant melanoma. *Br. J. Dermatol.*, **109**, 165.

CLARK, W.H. Jr, REIMER, R.R., GREENE, M.H., AINSWORTH, A.M. & MASTRANGELO, M.J. (1978). Origin of familial malignant melanomas from heritable melanocytic lesions: the BK mole syndrome. *Arch. Dermatol.*, **114**, 732.

DUBIN, N., MOSENON, M. & PASTERNAK, B.S. (1986). Epidemiology of malignant melanoma: pigmentary traits, ultraviolet radiation and the identification of high-risk populations. In *Epidemiology of Malignant Melanoma*. Gallagher, R.P. (ed.), pp. 56–75. Springer-Verlag: New York.

ELWOOD, J.M., WILLIAMSON, C. & STAPLETON, P.J. (1986). Malignant melanoma in relation to moles, pigmentation and exposure to fluorescent and other lighting sources. *Br. J. Cancer*, **53**, 65.

GREEN, A., BAIN, C., MCLENNAN, R. & SISKIND, V. (1986). Risk factors for cutaneous melanoma in Queensland. *Recent. Res. Cancer Res.*, **102**, 76.

GREEN, A., MACLENNAN, R. & SISKIND, V. (1985). Common acquired naevi and the risk of malignant melanoma. *Int. J. Cancer*, **35**, 297.

GREENE, M.H., CLARK, W.H. Jr, TUCKER, M.A., KRAEMER, K.H., ELDER, D.E. & FRASER, M.C. (1985). High risk of malignant melanoma-prone families with dysplastic nevi. *Annals Int. Med.*, **102**, 458.

HICKS, N., ZACK, M., CALDWELL, G.G. & MCKINLEY, T.W. (1985). Life-style factors among patients with melanoma. *South Med. J.*, **78**, 903.

HOLLY, E.A., KELLY, J.W., SHPALL, S.N. & CHIU, S.-H. (1987). Number of melanocytic nevi as a major risk factor for malignant melanoma. *J. Am. Acad. Dermatol.*, **17**, 459.

HOLMAN, C.D.J. & ARMSTRONG, B.K. (1984). Pigmentary traits, ethnic origin, benign nevi, and family history as risk factors for cutaneous malignant melanoma. *JNCI*, **72**, 257.

KLEPP, O. & MAGNUS, K. (1979). Some environmental and bodily characteristics of melanoma patients. A case-control study. *Int. J. Cancer*, **23**, 482.

KOPF, A.W., GOLD, R.S., ROGERS, G.S. & 4 others (1986). Relationship of lumbosacral nevocytic nevi to sun exposure in dysplastic nevus syndrome. *Arch. Dermatol.*, **122**, 1003.

KOPF, A.W., LAZAR, M., BART, R.S., DUBIN, N. & BROMBERG, J. (1978). Prevalance of nevocytic nevi on lateral and medial aspects of arm. *J. Dermatol. Surg. Oncol.*, **4**, 153.

KOPF, A.W., LINDSAY, A.C., ROGERS, G.S., FRIEDMAN, R.J., RIGEL, D.S. & LEVENSTEIN, M. (1985). Relationship of nevocytic nevi to sun exposure in dysplastic nevus syndrome. *J. Am. Acad. Dermatol.*, **12**, 656.

MACKIE, R.M., FREUDENBERGER, T. & AITCHISON, T.C. (1989). Personal risk-factor chart for cutaneous melanoma. *Lancet*, **ii**, 487.

NORDLUND, J.J., KIRKWOOD, J., FORGET, B.M. & 4 others (1985). Demographic study of clinical atypical (dysplastic) nevi in patients with melanoma and comparison subjects. *Cancer Res.*, **45**, 1855.

REIMER, R.R., CLARK, W.H. Jr, GREENE, M.H., AINSWORTH, A.M. & FRAUMENI, J.F. (1978). Precursor lesions in familial melanoma: a new genetic preneoplastic syndrome. *JAMA*, **239**, 744.

RHODES, A.R., SOBER, A.J., MIHM, M.C. Jr & FITZPATRICK, T.B. (1980). Possible risk factor for primary cutaneous malignant melanoma (abstract). *Clin. Res.*, **28**, 252A.

ROUSH, G.C. (1988). Chapter 6. Abnormal nevi, excess total nevi and melanoma: an epidemiologic perspective. In *Malignant Melanoma: Biopsy, Diagnosis and Therapy*, pp. 85–100, 191–195. Kluwer: Boston.

ROUSH, G.C., HOLDFORD, T.R. & SCHYMURA, M.J. (1987). Cancer risk and incidence trends. *Hemispheric*, 203.

ROUSH, G.C., NORDLUND, J.J., FORGET, B., GRUBER, S.B. & KIRKWOOD, J.M. (1988). Independence of dysplastic nevi from total nevi in determining risk for non familial melanoma. *Prevent. Med.*, **17**, 273.

SWERDLOW, A.J., ENGLISH, J., MACKIE, R.M. & 4 others (1986). Benign melanocytic nevi as a risk factor for malignant melanoma. *Br. Med. J.*, **292**, 1555.

ZAR, J. (1984). *Biostatistical Analysis*. Prentice-Hall Inc, Second Edition, pp. 323–325.


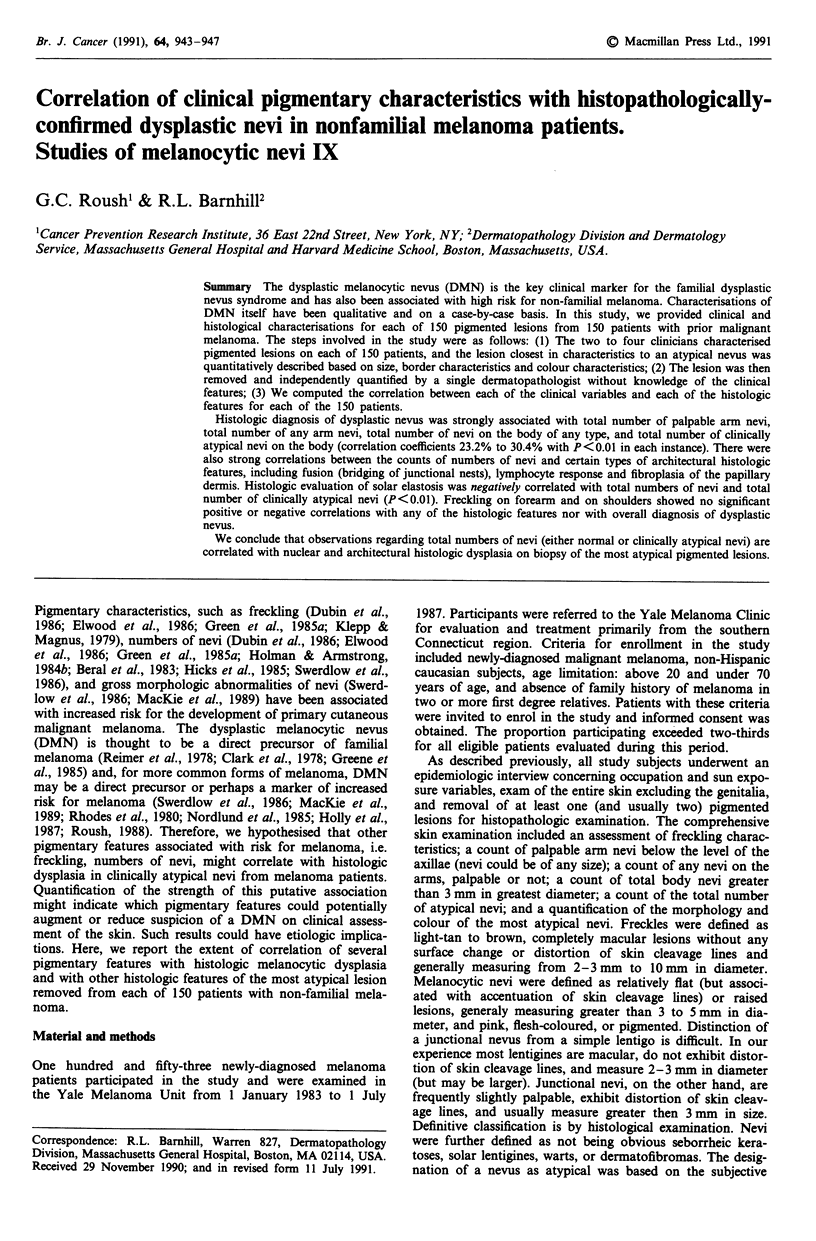

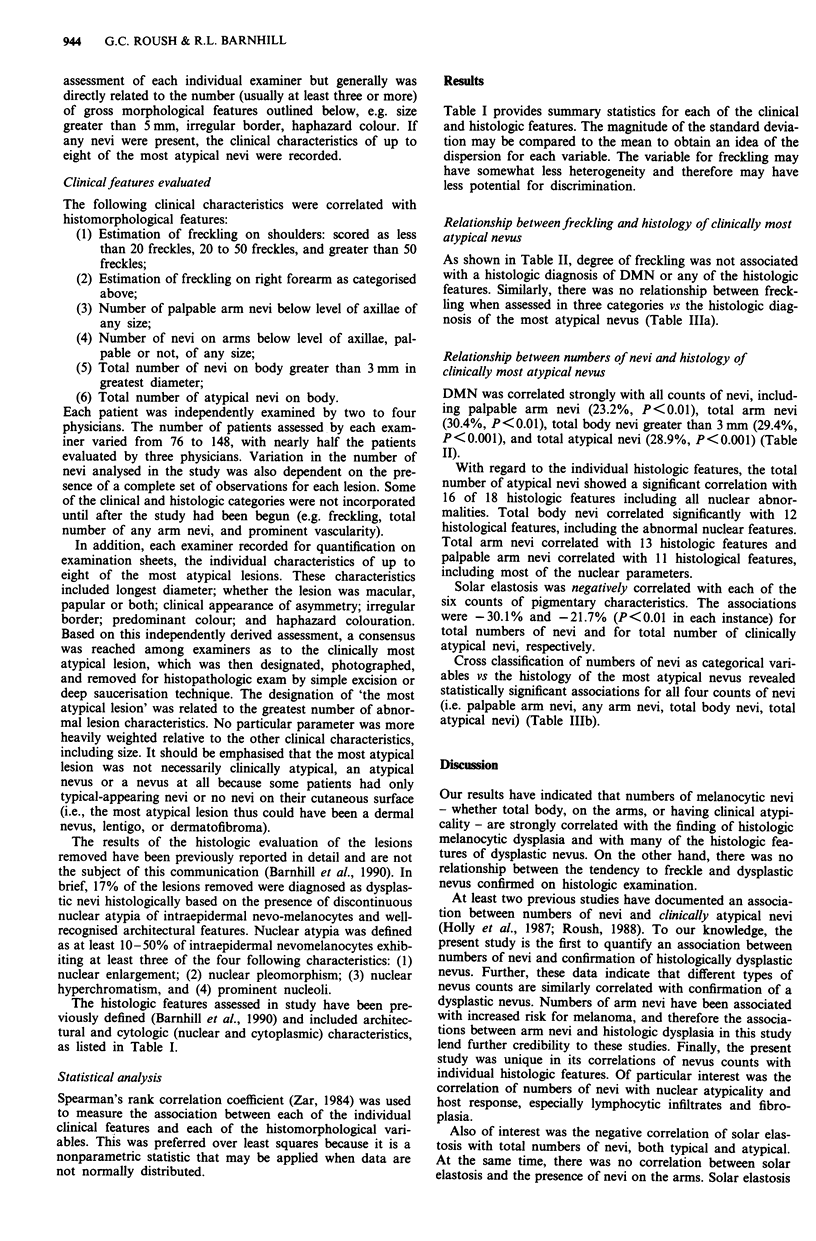

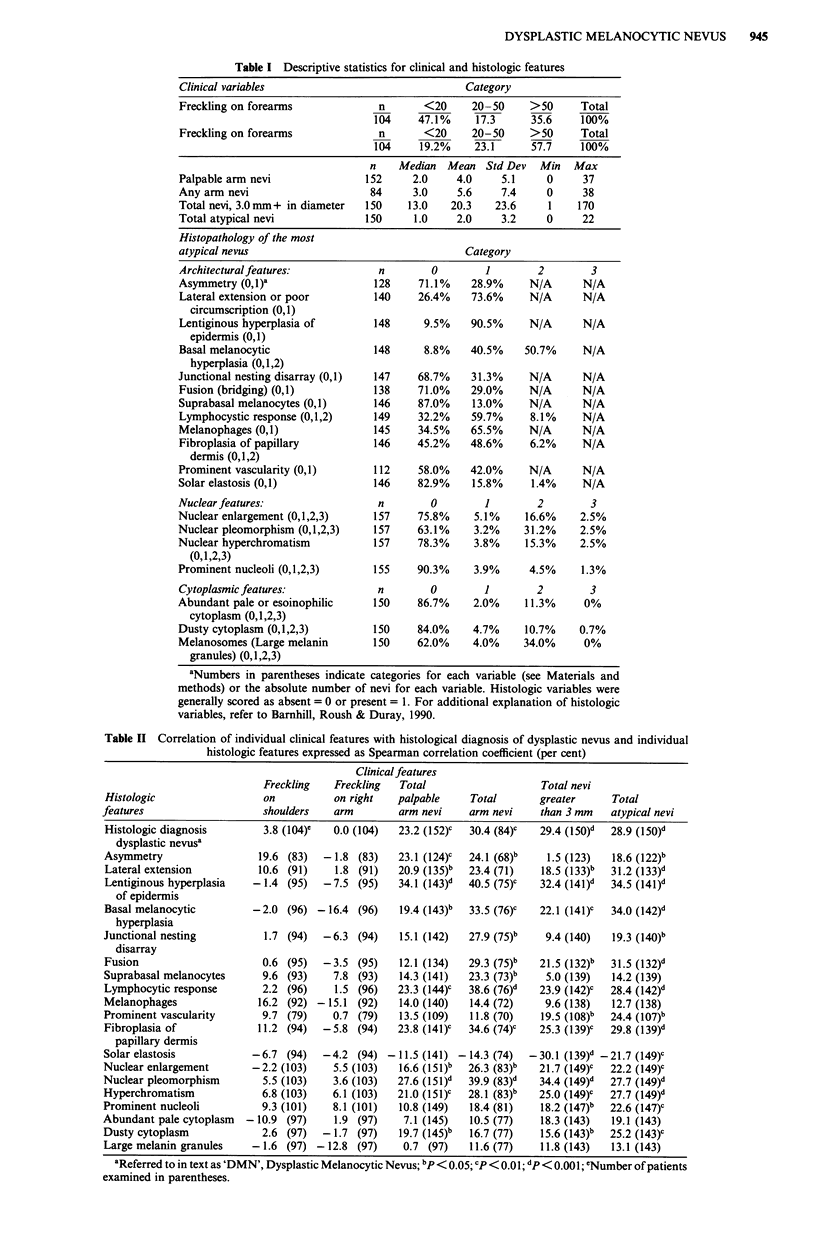

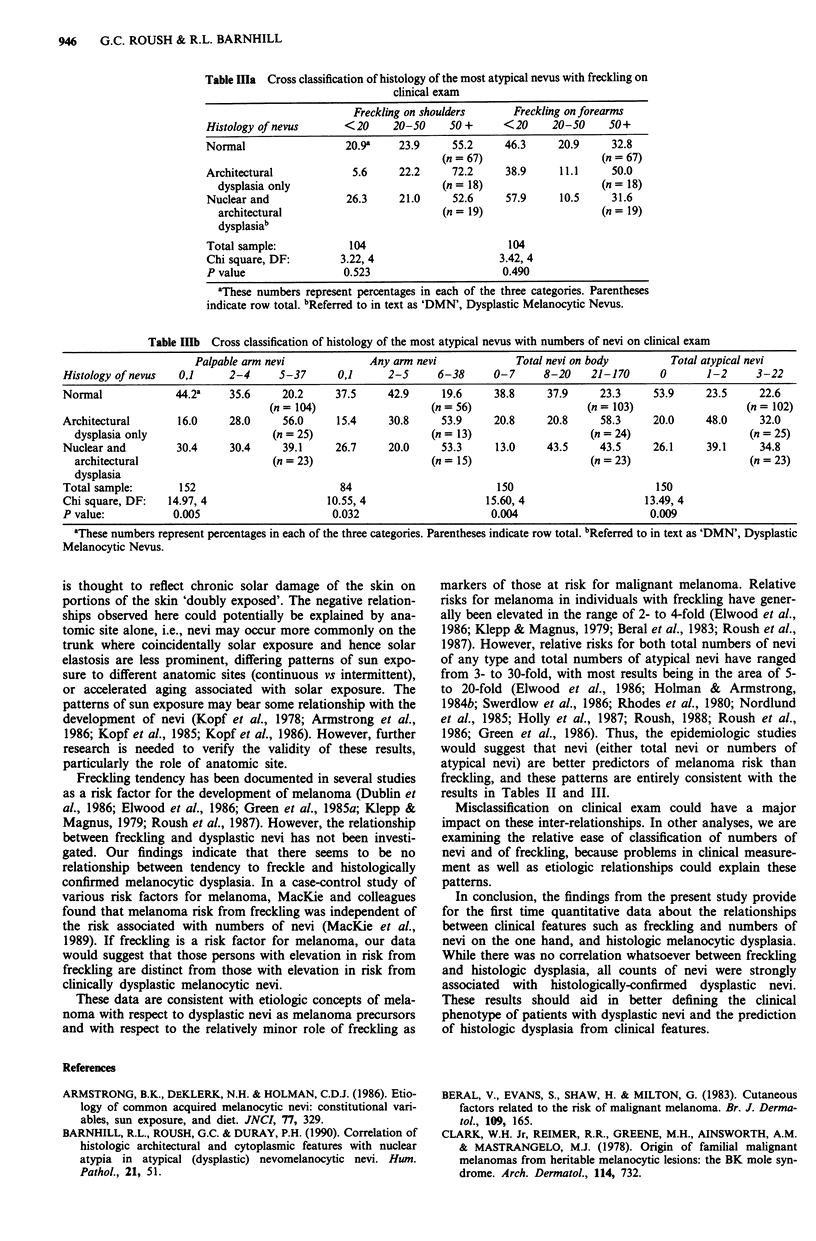

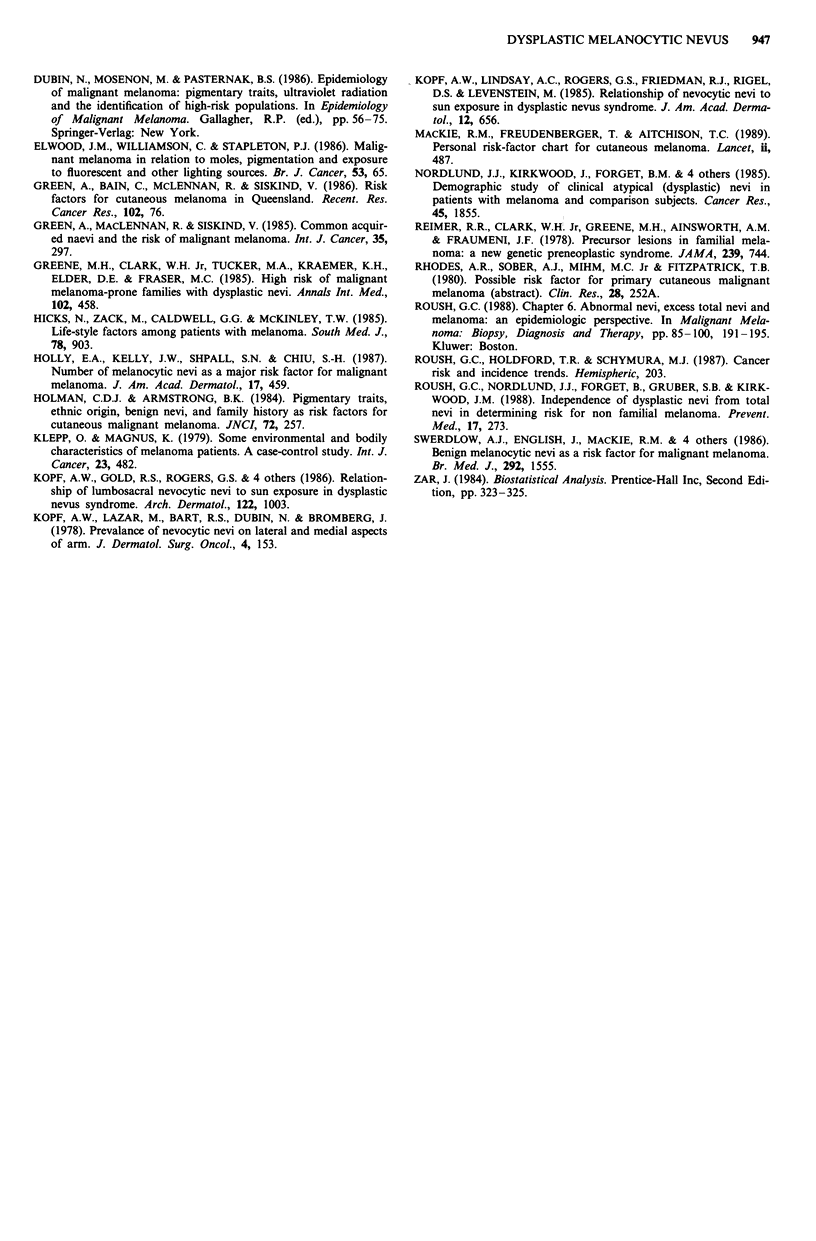


## References

[OCR_00649] Armstrong B. K., de Klerk N. H., Holman C. D. (1986). Etiology of common acquired melanocytic nevi: constitutional variables, sun exposure, and diet.. J Natl Cancer Inst.

[OCR_00654] Barnhill R. L., Roush G. C., Duray P. H. (1990). Correlation of histologic architectural and cytoplasmic features with nuclear atypia in atypical (dysplastic) nevomelanocytic nevi.. Hum Pathol.

[OCR_00660] Beral V., Evans S., Shaw H., Milton G. (1983). Cutaneous factors related to the risk of malignant melanoma.. Br J Dermatol.

[OCR_00665] Clark W. H., Reimer R. R., Greene M., Ainsworth A. M., Mastrangelo M. J. (1978). Origin of familial malignant melanomas from heritable melanocytic lesions. 'The B-K mole syndrome'.. Arch Dermatol.

[OCR_00673] Dubin N., Moseson M., Pasternack B. S. (1986). Epidemiology of malignant melanoma: pigmentary traits, ultraviolet radiation, and the identification of high-risk populations.. Recent Results Cancer Res.

[OCR_00680] Elwood J. M., Williamson C., Stapleton P. J. (1986). Malignant melanoma in relation to moles, pigmentation, and exposure to fluorescent and other lighting sources.. Br J Cancer.

[OCR_00684] Green A., Bain C., McLennan R., Siskind V. (1986). Risk factors for cutaneous melanoma in Queensland.. Recent Results Cancer Res.

[OCR_00689] Green A., MacLennan R., Siskind V. (1985). Common acquired naevi and the risk of malignant melanoma.. Int J Cancer.

[OCR_00694] Greene M. H., Clark W. H., Tucker M. A., Kraemer K. H., Elder D. E., Fraser M. C. (1985). High risk of malignant melanoma in melanoma-prone families with dysplastic nevi.. Ann Intern Med.

[OCR_00700] Hicks N., Zack M., Caldwell G. G., McKinley T. W. (1985). Life-style factors among patients with melanoma.. South Med J.

[OCR_00705] Holly E. A., Kelly J. W., Shpall S. N., Chiu S. H. (1987). Number of melanocytic nevi as a major risk factor for malignant melanoma.. J Am Acad Dermatol.

[OCR_00710] Holman C. D., Armstrong B. K. (1984). Pigmentary traits, ethnic origin, benign nevi, and family history as risk factors for cutaneous malignant melanoma.. J Natl Cancer Inst.

[OCR_00715] Klepp O., Magnus K. (1979). Some environmental and bodily characteristics of melanoma patients. A case-control study.. Int J Cancer.

[OCR_00720] Kopf A. W., Gold R. S., Rogers G. S., Hennessey N. P., Friedman R. J., Rigel D. S., Levenstein M. (1986). Relationship of lumbosacral nevocytic nevi to sun exposure in dysplastic nevus syndrome.. Arch Dermatol.

[OCR_00725] Kopf A. W., Lazar M., Bart R. S., Dubin N., Bromberg J. (1978). Prevalence of nevocytic nevi on lateral and medial aspects of arms.. J Dermatol Surg Oncol.

[OCR_00730] Kopf A. W., Lindsay A. C., Rogers G. S., Friedman R. J., Rigel D. S., Levenstein M. (1985). Relationship of nevocytic nevi to sun exposure in dysplastic nevus syndrome.. J Am Acad Dermatol.

[OCR_00736] MacKie R. M., Freudenberger T., Aitchison T. C. (1989). Personal risk-factor chart for cutaneous melanoma.. Lancet.

[OCR_00741] Nordlund J. J., Kirkwood J., Forget B. M., Scheibner A., Albert D. M., Lerner E., Milton G. W. (1985). Demographic study of clinically atypical (dysplastic) nevi in patients with melanoma and comparison subjects.. Cancer Res.

[OCR_00747] Reimer R. R., Clark W. H., Greene M. H., Ainsworth A. M., Fraumeni J. F. (1978). Precursor lesions in familial melanoma. A new genetic preneoplastic syndrome.. JAMA.

[OCR_00756] Roush G. C. (1988). Abnormal nevi, excess total nevi, and melanoma: an epidemiologic perspective.. Cancer Treat Res.

[OCR_00772] Swerdlow A. J., English J., MacKie R. M., O'Doherty C. J., Hunter J. A., Clark J., Hole D. J. (1986). Benign melanocytic naevi as a risk factor for malignant melanoma.. Br Med J (Clin Res Ed).

